# Women with disabilities’ experiences of intimate partner violence: a qualitative study from Sweden

**DOI:** 10.1186/s12905-023-02524-8

**Published:** 2023-07-20

**Authors:** Cartrine Anyango, Isabel Goicolea, Fredinah Namatovu

**Affiliations:** 1 Department of Epidemiology and Global Health, Umeå University, Umeå, Sweden; 2 Centre for Demographic and Ageing Research (CEDAR), Umeå University, Umeå, Sweden

**Keywords:** Intimate partner violence, Reflexive thematic analysis, Women with disabilities, In-depth interviews

## Abstract

**Background:**

Intimate Partner Violence (IPV) is a prevalent form of gender-based violence affecting one in three women globally. It is also a preventable cause of ill-health, disability, and death. Current research suggests that women with disabilities are at a significantly higher risk of experiencing violence throughout their lifetime. They are almost twice as likely to experience violence compared to men with disabilities or men and women without disabilities. Additionally, they experience higher rates of all types of violence. This increased vulnerability may be due to factors related to disability such as dependence on others for support, mistrust, and social and physical isolation. Although there is existing research on IPV against women in general, there is limited knowledge on IPV against women with disabilities. To address this gap in knowledge, this study aimed to explore women with disabilities’ perceptions and experiences of being victims/survivors of IPV in Sweden.

**Methods:**

This was a qualitative study conducted through in-depth interviews with eleven women with disabilities. The participants were aged eighteen years upwards. The collected data was analyzed using reflexive thematic analysis with a constructivist epistemological standpoint.

**Results:**

We developed four themes. Theme one: “multiple abuse by multiple abusers, over time,” describes the participants’ experiences of various types of violence from different perpetrators for prolonged periods. Theme two: “psychological abuse—harmful, but neglected and difficult to prove,” explains how women with disabilities’ perceive psychological abuse as harmful, but not given the same level of seriousness as physical violence. It also expresses the difficulties they encountered in providing tangible evidence to prove instances of psychological abuse. Theme three: “abuse does not end with separation,” highlights how abuse can continue beyond separation/divorce. Theme four: “surviving abusive relationships” describes the different and evolving ways the participants used to navigate their abusive relationships.

**Conclusions:**

Women with disabilities face all forms of abuse. They find it challenging to prove psychological abuse, and the system is inadequate in addressing its harm. The abuse also continues after separation or divorce. The support system should consider the needs of women with disabilities who experience violence, both during and after the abusive relationship. Service providers should be better equipped to detect and handle all types of IPV, especially psychological abuse.

**Supplementary Information:**

The online version contains supplementary material available at 10.1186/s12905-023-02524-8.

## Background

The World Health Organization (WHO) defines intimate partner violence (IPV) as a “behavior by a current or ex intimate partner that causes physical, sexual or psychological harm, including acts of physical aggression, sexual coercion, psychological abuse and controlling behaviors” [1]. IPV is a gendered public health and social problem that results in significant health, economic, and social expenditure [2–4]. IPV affects one in three women globally and is a leading type of gender-based violence, yet a preventable cause of ill-health, disability, and death [5]. Certain groups of women are at increased risk of IPV and face more barriers to accessing support; one such group represents women with disabilities. Women with disabilities face almost double the lifetime risk of violence compared to women without disabilities [6].

Worldwide, there are approximately 250 million women with disabilities, while in Europe about 16% of women are estimated to have disabilities [7]. Recent research, including a systematic review, reported that women with disabilities, experience higher rates of all kinds of violence than either men with disabilities or men and women without disabilities [6, 8–11].

In Europe, about 40% of women with disabilities have experienced violence, including IPV [7]. Elsewhere, according to a United States’ national data, women with disabilities are at greater risk of all forms of violence [12]. Meanwhile, in Africa, a cross-sectional survey in Uganda detailed that women with disabilities were more likely to experience lifetime physical and emotional abuse than those without disabilities [13], and a recent study in Tanzania found associations between specific types of disabilities and elevated risks of IPV exposure [14].

While women with disabilities experience abuse from family members and medical personnel, among others, the most common perpetrators of violence against women with disabilities are male (current or former) intimate partners [11, 15, 16]. Women with disabilities are more often subjected to severe forms of physical violence, sexual abuse, and psychological abuse than women without disabilities [6, 10–12, 17]. The literature suggests the existence of notable differences in the duration and forms of violence. Women with disabilities have been reported to experience multiple forms of violence (psychological, physical, and sexual) and for longer periods of time than women without disabilities [18, 19]. In addition, women with disabilities may also experience disability-related forms of violence for extended periods of time and from multiple abusers within disability-related settings, such as special residential areas, hospitals, clinics, vehicles, or schools, while the need for personal assistance increases vulnerability to abuse [19].

A review of the literature by Smith (2008) provided some explanations behind the higher rates of IPV among women with disabilities. For example, those with physical disabilities may need help with replacing the battery on a wheelchair, toilet operations, or dressing, among other intimate actions. This kind of dependence may lead to perpetrators taking advantage of the women’s vulnerability for instance, demanding a kiss before assisting, withholding medication, and removing the battery from the wheelchair are some examples of disability-related abuse experienced by women with disabilities [6]. Furthermore, other factors common to many women exposed to IPV, such as economic dependence on their partners or lack of information about existing resources, can hinder women with disabilities’ ability to leave abusive relationships.

Moreover, women with intellectual disabilities may encounter challenges in identifying instances of violence, and they may face disbelief or lack of support from service providers when they attempt to report such incidents [6, 11, 20]. Since intellectual disabilities affect cognitive development and functioning, the degree of impairment can impact an individual’s capacity to recognize and understand abuse.

### IPV in Sweden

Studies conducted in Sweden have reported varying rates of violence experienced by women. For example, Anderson (2015) found that 14% of women had experienced physical violence in their lifetime [21], Ahnlund reported a 6% prevalence of violence experienced in the past year among older women [22], and Lövestad reported that 41% of women had been subjected to controlling behaviors or emotional abuse in their lifetime [23].

Generally, in Sweden, we know little about the IPV experiences of women with disabilities. Olofsson et al. found that women with visual disabilities are at greater risk of exposure to both physical and psychological violence than women without such disabilities [24]. Other available knowledge about IPV relates to women in general, statistical information, or numbers and studies reporting the perspectives of others but not of the women with disabilities who have experienced IPV themselves. For instance, quantitative studies exist on IPV against women in general, focusing on screening for IPV prevalence and determinants [25], prevalence of IPV in association with depression [26], and IPV-related help-seeking [27, 28]. Qualitative studies have explored the IPV experiences of women in general and the care provided for them [29]; women’s perceptions and experiences of leaving abusive relationships [30]; and women’s discernible emotion work in the context of leaving abusive men [31].

### Study rationale and aim

Just like in other regions of the globe, extensive qualitative research has been conducted in Sweden and other Nordic countries focusing on IPV against women in order to understand the beliefs, behaviors, and norms related to IPV against women [32]. From the qualitative studies conducted in Sweden with women victims of IPV, we have learned that women experience IPV in all its forms, that they face difficulties in accessing help and support, and that they lack trust in service providers, which may further discourage them from reporting abuse [29, 33]. Existing qualitative research further shows that women feel ashamed of being victims of violence [30] and that experiences of violence and abuse continue over time, from the past to the present, and frame beliefs about the future [34].

However, very few studies, if any, have specifically explored the perceptions and experiences of women with disabilities as victims or survivors of IPV. As described by the WHO and program for appropriate technology in health (PATH), qualitative research can help to “understand cultural norms, beliefs, and behaviors or to capture and analyze complex motivations” [35]. Therefore, it is important to understand the experiences of women with disabilities as expressed by the women themselves because research shows that they are at increased risk [6, 10, 17], yet there is a scarcity of studies exploring their experiences [36]. Understanding how women with disabilities perceive and experience IPV is vital for the better identification of IPV against them and for developing adequate strategies and targeted support for them.

To fill this knowledge gap, this study’s aim was to explore women with disabilities’ perceptions and experiences of being victims/survivors of intimate partner violence in Sweden.

## Methods

This is a qualitative study, using in-depth interviews with women with disabilities who have been victims/survivors of IPV. The study adopted a constructivist epistemological standpoint—which recognizes that knowledge is shaped by social and cultural contexts, and that researchers’ own perspectives can influence how they interpret data. It acknowledges that reality is not a fixed concept but rather shaped by our own perceptions and experiences [37, 38]. We acknowledged our own subjectivity and diverse backgrounds to understand how our assumptions could influence the process of analyzing data. At the same time, we put focus on participants perspectives, perceptions and experiences and the ways in which they construct meaning from their own experiences.

### Procedures

#### Participants and recruitment

We used purposive sampling [39, 40] to select participants. The study was restricted to women who had a disability, aged over 18 years, and had experienced any form of violence from an intimate partner. We recruited participants by advertising the study through disability organizations’ membership magazines, social media, national radio, and women’s shelters. Thirteen women expressed interest to participate and contacted the research team, but only eleven were interviewed. The other two were not interviewed because one had time constraints and one with a hearing impairment did not have an interpreter. It is important to emphasize that during the interviews, participants were not specifically asked about the specific nature of their disabilities, nevertheless, many voluntarily shared this information even though they were not required to. During data analysis, we categorized their disabilities based on their narrations as they recounted their experiences. According to our categorization, among the eleven participants, three had physical disabilities, seven had intellectual disabilities, and one participant did not explicitly specify the type of her disability while sharing her experiences. Participants originated from diverse regions of Sweden. Their ages ranged between 25 and 55 years. Five were single at the time of the interview, three were separated/divorced, one was married, and two did not disclose their marital status at the time of interview. All the participants were in a heterosexual relationship-when sharing their experiences of IPV, they referred to male intimate partners. Four had children. Five were working, albeit not full time.

### Data collection

This study is a component of an ongoing research project that aims to examine the accessibility and utilization of IPV services. We conducted eleven in-depth interviews, using a semi-structured interview guide. The interview guide encompassed questions grouped into four broad categories: (1) accessibility and contact with IPV support and services, (2) quality of IPV support services and suitability, (3) competence development and recommendations, and (4) COVID-19 and IPV. For the purposes of this study, we primarily focused on materials related to the first category. For a detailed list of specific questions posed to the participants, please refer to the interview guide provided in additional file 1.

A member of the project team conducted the interviews in Swedish, the participants’ preferred language. The interviews were conducted virtually due to the COVID-19 pandemic’s restrictions. Cognizant of the participants’ different disabilities, the interviewer moved at their pace. For instance, some interviews took longer because participants had to pause between questions or became distracted by other things. On such occasions, the interviewer engaged them in other topics until they were ready to resume the interview. The interviews lasted between 56 and 100 min, were recorded, transcribed verbatim, and then translated into English. Participants’ names were pseudonymized.

### Data analysis

We used reflexive thematic analysis (RTA) [41]. RTA is a theoretically flexible interpretive approach to qualitative data analysis that highlights the researcher’s active role in knowledge production [41]. We followed RTA’s six-phase process: [1] familiarization with the data and writing familiarization notes, [2] generating initial codes, [3] generating initial themes from the coded data, [4] reviewing potential themes, [5] refining, defining, and naming themes, and [6] writing/producing the report [42–44]. The six phases are organized in chronological order; however, we conducted the analysis in an iterative format, moving back and forth through the phases as we are aware that qualitative data analysis is not a unidirectional process [42, 43]. The first author spent a substantial amount of time in the first phase of analysis—familiarization with the data—by repeatedly listening through the audio recordings, reading the transcripts, and writing notes. The transcribed dataset was then exported to MAXQDA, a qualitative data analysis software package, to support the development of codes. All the authors read, coded, and discussed several transcripts teasing out recurring patterns. The first author then finalized the coding process. Even though the coding was done on MAXQDA, all the codes were afterwards transferred to Microsoft Excel, where we performed the thematization. We interactively and iteratively scrutinized and grouped the codes as appropriate to generate potential themes. Then, we reviewed these potential themes, moving back and forth between the transcripts, codes, and potential themes, and created a thematic map to aid in piecing together our narrative. Lastly, we reviewed the potential themes; refining, renaming, and collapsing some of them, resulting in the final four themes that we present under the results section.

### Ethical considerations

This study obtained approval from the Swedish Ethical Review Authority [J. Reg no. 2019–05249].

To ensure participants’ safety, we pseudonymized their names, personal information (name, address, etc.) was not included in the transcribed data, and the recorded videos/audios were stored separately from the transcribed data. To enhance voluntary participation, all the participants were informed that they had the right to withdraw at any point during the interview process, and they provided both written and oral informed consent. To guarantee participants’ confidentiality and anonymity, they made the decision to either have the video recorder on or off during the virtual interviews. Finally, following the ethical board’s guidelines and due to the sensitivity of the topic under study and the possibility that it might evoke negative emotions and stress, the interview information and invitation letter to the participants contained details of the project’s contact person in the community, who was familiar with IPV services and able to provide the participants with guidance and referral to available help and support as necessary.

### Methodological considerations and reflexivity

We strived to guarantee the quality of our study by following Braun and Clarke’s guidelines on ensuring quality when conducting a qualitative study [41, 43]. Firstly, we have specified the type of thematic analysis we used and did not assume that all thematic analysis consists of “one approach” [43]. We used reflexive thematic analysis, a method that considers the researchers’ values and subjectivity throughout the process [43]. Secondly, we conducted coding inductively without following any coding framework and iteratively developed our themes, which were the final product of the data coding process [43]. In addition, we used both semantic (codes closer to the data) and latent (interpretative codes) coding, without prioritizing one over the other [41, 42]. Throughout the analysis, we conceptualized a code as a unit of analysis and used codes to generate themes. The generation of themes took place through an iterative process during the analysis work; this was an active and creative process engaging all the authors. We documented the entire analysis process, from the generation of codes to thematization, to ensure that the origins of the themes were clear and traceable to us through the process, and we have used quotes to enhance clarity for readers.

One of the fundamental aspects of RTA is reflexivity—critical reflection upon the researcher’s role in knowledge production [41]. The guiding principle we adopted was to reflect on the women with disabilities’ own accounts of their perceptions and experiences of IPV as authentically as possible, while at the same time reflexively taking into consideration the influence of our own interpretations as authors [41, 42]. As described by Berger, reflexivity is “the process of a continual internal dialogue and critical self-evaluation of the researcher’s positionality as well as active acknowledgement and explicit recognition that this position may affect the research process and outcome” [45]. We repeatedly discussed our different understandings of the study, the participants, and the data, which was helpful in questioning our pre-understandings. One of the authors (FN), the principal investigator, was more familiar with the entire material from the beginning as she had conceptualized the project. The first author (CA) interacted extensively with the data even though she had not taken part in the preliminary stages, while the other author (IG) participated in the preliminary stages and brought to the project her wide experience in qualitative research, concepts, and the topic under study. In addition, the three of us represent different disciplines and backgrounds. These aspects and diversities contributed to interesting discussions during the research process, leading to constructive questioning of our ideas and pre-understandings, which in turn led to a more nuanced analytical process. We also conducted peer-debriefing with other team members not included as authors, thus allowing for further scrutiny of our pre-understandings.

However, our study has limitations. Due to logistical constraints, we did not share our preliminary results with the participants, which might have further illuminated other aspects. Another limitation was not being able to include the experiences of other women who face multiple forms of oppressions specifically from an intersectional perspective. This includes women who identify as queer, immigrant women, and women of color. Additionally, we acknowledge that women with the most severe disabilities may be the most vulnerable, but we were unable to reach them in our study. Furthermore, when it comes to disability, examining various disabilities within the same study may have hindered our ability to gain a deeper understanding of any specific disability.

## Results

Altogether, we developed four themes that provide insight into the perceptions and experiences of IPV among women with disabilities who have been victims or survivors of IPV in Sweden.

### Multiple abuse by multiple abusers, over time

He never hit me in the face or body, but he is very harsh, manipulative, domineering, abusive, uses ugly words, taunts, and now financial violence […] I had been dependent on my ex-husband a lot due to my hearing impairment he took over everything about finances in general. (Ebba)

Yes. I met a man I became pregnant with. He subjected me to attempted rape. (Ulla)

For him [my ex], it [helping and supporting me] wasn’t important. I have a hearing aid, so it gets hot, and it itches. When I got stressed, it got even worse as I fiddled so much and couldn’t get it in, then it went too far into my ear […]. At night, I screamed in pain, I said to him: “Can you call [the healthcare center]? It hurts so much,” but he said: “No, it will be fine.” I said: “Call! help me.” He didn’t. (Ebba)

The three quotes above are examples of how the women with disabilities in this study were exposed to multiple forms of abuse, comprising financial, sexual, and psychological forms. Financial abuse entailed, for example, intimate partners taking advantage of women’s disability status to bypass them, navigate the system, access their disability allowance, and “empty the accounts” without their consent. As Ebba’s first quote shows, partners took advantage of the women’s dependence on them as an avenue to control their finances.

Although the women in this study experienced several forms of abuse from multiple abusers, psychological abuse, and neglect were especially common across their narrations, as the second quote from Ebba shows. Psychological abuse was exerted through gaslighting, invasion of privacy and violation of personal belongings. Neglect, on the other hand, was through refusal to help. The women interviewed vividly described instances of being subjected to “wrong treatment,” enduring “threats and intimidation,” being left alone without support at home and labelled as incapable of fulfilling their responsibilities due to their disabilities.

As well as experiencing different types of violence, the women in this study also experienced abuse from different types of abusers:

My god man treated me bad and exposed me to debts. After that god man, I got a 75-year-old man. He treated me bad too. He was so controlling […]. It didn’t work at all. It was completely crazy. (Ulla)

When I was 16 years old, it was a friend of the family who sexually abused me […] and then later a male staff member told me to undress and so on, in my residence, and we were alone in the room, forcing me to hug several times. (Jenny)

As Ulla describes in the quote above, her “god man,” who is an individual assigned by social services to assist people with disabilities in making decisions and navigating challenges related to their disabilities, can instead become controlling, inflicting bad treatment, abuse, and violence. The women interviewed were also exposed to violence from a wide array of people with whom they had close relationships, such as partners, ex-partners, and parents. Other people, such as friends and colleagues who accessed these women’s intimate spaces, also overstepped their roles, took advantage of these women’s disabilities, and ended up perpetrating abuse, as Jenny described in the quote above.

Finally, the women described how their experiences of violence and abuse occurred over time.

My mom did it. It had been, not punches, but slaps, being pushed into furniture very hard. When I was little, she held my arms so tightly that I got bruises. It stopped when I got older and could resist. (Pia)

In the above quote, Pia described having experienced abuse from her mother, whom she expected to be her source of help and support. She was not an exception; among the participants, some women talked about experiences of violence that started when they were children and continued throughout their youth and adulthood.

In summary, the women described experiences of multiple forms of violence by multiple perpetrators and for prolonged durations. Among the different forms of abuse, psychological abuse was perceived as particularly harmful, as well as being “hard to prove.” We turn to this in the next theme.

### Psychological abuse—harmful, but neglected and difficult to **prove**

I guess I could go to the police with them [episodes of psychological abuse], but as many say, it usually doesn’t get picked up or it’s not taken further and so on, and I’ve heard others who’ve said one should perhaps not compare and say that one [form of abuse] is worse, but there are some who’ve been more physically abused than I faced in that relationship, for that person it has just been taken seriously […] Yes, the physical, it’s like, it still heals like relatively often, maybe a little faster than the psychological, which can often be there. (Sofia)

In terms of pervasiveness, the interviewed women perceived psychological abuse as more harmful than physical violence, which they said, “heals faster.” Psychological abuse was described as “invisible” and as having a greater impact on an individual’s health and well-being, as Sofia explained in the quotation above. In addition, psychological abuse was perceived as brutal and requiring “more help” because, in Ebba’s words, it makes one “not want to live.” Despite the perceived pervasiveness and severity of psychological abuse, the women in this study also perceived psychological abuse as neglected and/or not taken seriously by the services and support institutions. Services and support institutions here refer to the police, healthcare, and social services.

When it came to my relationship, I never sought any support because he never hit me. So, I never had concrete examples. I knew it shouldn’t be like that. But he never crossed a line, to become clear for me to report [….] then I just realized that I shouldn’t tell everything. This is the typical situation that I hear many people are in. In my case, it was: “he never hits [you], so what’s the problem?” He just got a little angry. Yes, he broke the wall next to my head, but he didn’t hit me. He hit the wall. (Mona)

When can you report to the police? Sometimes I said: “Beat me, so I can report you to the police,” but he never did. He knew it. That was the hardest part of it all. (Ebba)

The above quotes describe how psychological abuse is not considered very important, while hitting, beating, and any other form of physical injury that is clearly visible was/is considered serious and qualified as having “crossed the line” and, most importantly, becomes a motivation for seeking support. If violence was not physical, there was the perception that it would not be taken seriously by the support institutions that had the liberty to decide whether a case had proof of abuse or not. This, in turn, discouraged the women from seeking help when they had not experienced that type of violence, as shared by Mona in the quote above. The frustration of psychological abuse not being taken seriously due to the lack of physical proof led the participants to seek such proof and question whether they should actually seek physical abuse. From Ebba’s quote above, we want to argue that this (asking to be beaten) reflects the frustrations of psychological abuse requiring proof.

According to these women’s perspectives, Sweden lacks laws to prosecute psychological abusers; thus, the support institutions do not have legal frameworks consisting of laws and rules to punish such abusers. But it was not only a matter of lacking laws to punish this type of violence; the participants also perceived society at large as (in Lena’s words) “talking a lot about the physical violence but forgetting to talk about the psychological abuse which [society] considers quite a taboo.” They called for strategies to start a discussion on the importance of taking psychological abuse seriously as an important issue and not as a “taboo” as it is now viewed.

Even though they were faced with suspicion and the need to prove psychological abuse, all the participants had managed to quit their abusive relationships. However, the violence continued after leaving their abusers, as we illustrate in the next theme.

### Abuse does not end with separation

We have children together; things must be bought. Then I suggested to him that we can pay together and then I pay a percentage of my salary. “No, 50/50,” he insists, “I have no obligation to support you.” “It’s not about me, it’s about the children,” I said. He doesn’t understand anything. He won’t stop the harassment. (Ebba)

Even though we’d agreed that I should have our boy, he’s kept the boy and gone underground. (Ingrid)

All the women in this study had ended the abusive relationships, either through separation or divorce. However, despite the physical separation, they still faced ongoing psychological abuse from their former intimate partners. Those with co-parenting responsibilities and unstable financial situations endured continued financial frustrations and control, as the abusers exploited these circumstances to exert power and make them feel powerless. For instance, even though it was known that these women only worked part-time, their ex-partners insisted on a 50/50 financial arrangement for childcare as mandated by the law. However, it is worth noting that the same law allows parents to make special arrangements based on their mutual agreement to cater to their children’s needs. This situation gave the abusers more power to continue their financial control and intimidation, as described by Ebba above.

Ex-partners made co-parenting difficult and continued to exert control and intimidation. As Ingrid stated above, she was denied the right to be with her child even after an agreement during separation, that co-parenting would be made possible as specified by the law. The perpetrators used co-parenting as an avenue to continue exercising psychological abuse.

Some ex-partners continued to take advantage of the women’s disabilities. For example, they made it difficult to sustain a conversation. In Ebba’s case, as she shared her personal experience below, her ex-husband continued to take advantage of her hearing impairment, dominated the conversation, and never gave her an opportunity to be heard.

So, I called him, and it was almost impossible to talk to him. It’s not possible to talk on the phone with him. He totally runs me over. (Ebba)

The ex-partners without a disability positioned themselves as the ones with a superior voice and used this to exert power and control through communication, illustrated by their domineering manner during conversations. Other ex-partners could not handle rejection. They perceived that a disabled woman should not leave a relationship, and thus turned to digital abuse; sending out embarrassing and untrue online texts to close networks indicating that she was “single,” “looking for a boyfriend” (Jenny) or sharing her “phone number with other guys” (Jenny).

Having experienced multiple forms of violence from multiple abusers for prolonged periods of time, faced with suspicion and the need to prove violence, managing to leave their abusive relationships, and then still having to endure abuse even after separation, the women in this study retrospectively recollected how they had coped, including attempts to resist such abusive partners. These women were doing something about their situation, both during and after the abusive process, which we explain in the next theme.

### Surviving abusive relationships

I became more and more isolated because I didn’t want to tell [anyone]. I thought it was embarrassing. I had also moved from my city to this guy’s city. Then it was even more that I didn’t want to show [it], I didn’t want to be seen as a failure and that I would have to move back to my city. (Mona)

When I ended up in deep depression, I found tools to process myself. These were the tools I was looking for online as well as, what does a healthy relationship look like? Is it me who’s crazy or this person [the perpetrator] who does things to me when I’ve said, “don’t do that,” and no one else knows that I’ve told him not to do those things? (Lena)

In hindsight, the women narrated how they had survived and navigated through the violent and abusive environment. Their approaches to surviving violence also changed along the way; from their current situation, where they had left their abusive partners (all the participants were separated or divorced at the time of the interviews), they reflected upon the different ways in which they had tried to survive the violence.

One way was to isolate themselves and to seek explanations and reasons for it. This way of surviving violence was related to the shame they felt about being a victim, a way to try to maintain appearances in front of others. Isolation was a way to avoid sharing abusive experiences because it was seen as “embarrassing,” as being a “failure” and different within society, as Mona described her personal experience in the quote above. This way of surviving took a toll on them because they had to continuously limit themselves, the activities they engaged in, and their social contacts. Retrospectively, these participants regretted such moments of isolating themselves and trying to endure the violence. As Ebba put it: “You blame yourself; how did I even allow it…. I’m still ashamed of how I could live with such a man.”

One other way that they survived the violence was by trying to make sense of their situation. They began questioning and comparing information on their own to help them deal with the stress and trauma. They took the initiative to search for on-line resources to support their healing and to prevent themselves from ending up in similar abusive situations again. These resources included information on tools to process self and examples of what a healthy relationship should look like as Lena shared in her quote above.

The women also mentioned a different way of surviving violence, which was through finding an escape from the space of violence, by engaging in activities they considered interesting that relieved the stress. Such endeavors included “writing music to let it flow out as such writing made me feel safe” (Sofia); or writing down the abusive experiences in a bid to just let it all out and get some relief. “Sometimes I went up and wrote a page and a half, just to write it down” (Ebba). Alcohol and other drugs were also a source of relief: “I knew I was drinking way too much. There has never been any doubt that I was aware of it because I used it as incorrect self-medication” (Mona).

Some of the factors that the women described as having enabled them to step up and leave their abusive relationships included support from friends and family, having had enough, doing it for the children’s sake, and having shared experiences with other abused women who had since left their abusive relationships. For instance, Ebba shared her personal experience of how she left the abusive relationship for the sake of her children:

I wanted us to be a family. I wanted us to. […] I didn’t want to divorce for the sake of the children, I protected them. But that wasn’t right, it was wrong. There was more violence. Then I realized that it wasn’t helping my children. I would perish, and the children would not feel good about seeing us use violence and abusive words.

## Discussion

This study has shown that women with disabilities face multiple forms of abuse, including sexual, financial, physical, and psychological. The perpetrators of abuse are diverse, and the durations of these experiences is often prolonged. The findings highlight that the participants perceived psychological abuse as being overlooked by the support institutions, placing the burden of proof on the victims themselves. This form of abuse was described as more pervasive than physical abuse. This study’s results further indicate that leaving the perpetrator did not necessarily end the abuse; it continued beyond separation. To endure and survive the difficulties encountered during their abusive relationships, the women in this study turned to seeking out explanations and questioning the spiteful experiences they were going through, finding escape through other stress-relieving actions, and digging for information to make sense of their situation. Leaving the abusive relationships was facilitated by the support of friends and family, having had enough, doing it for the children’s sake, and drawing strength from the shared experiences of other women who had also left abusive relationships. We unpack these findings using the following topics.

### Disability expands the intimate arena

Women with disabilities may require support from different people to access resources and meet their daily needs. Thus, disability makes them more dependent on their close relations, which, unfortunately, leads to more vulnerability. In the present study, having more people occupying these women’s intimate spaces meant having available help and support, but it also increased the risk of exposure to more perpetrators. Increased dependence on abusers for care and assistance, and social isolation have been reported as among the reasons for increased vulnerability and higher levels of abuse experienced by women with disabilities [6]. Our results show that, when it comes to IPV against women with disabilities, the concept of intimacy becomes expanded beyond the typical definition of current or former intimate partners. Having a disability expanded the definition of what an intimate relationship entailed. Participants experienced violence from other people who were close to them primarily for help and support reasons. Here, could the Swedish concept of violence within close relationships seem like a better fit? Violence within close relationships includes not only current and former intimate partners as potential perpetrators, but also the wider array of people with whom women with disabilities may be in close relationships. However, this concept has been criticized for its gender-neutrality; it downplays the gendered nature of IPV and leaves out the power imbalance and inequalities between men and women [46]. As Boyle expresses it: “naming practices make more or less visible who is doing what to whom, and foreground differing sets of connections” [47]. In the present study, for instance, on the one hand, using the term ‘intimate partner violence’ could leave out violence perpetrated by other people close to the victim, as intimacy expands when it comes to women with disabilities, but, on the other hand, using “violence in close relationships” could downplay the gendered nature of this type of violence.

### (In)visibility, (un)provability, and (il)legibility of psychological abuse

In this study, participants were faced with the heavy burden of proving that they were victims of psychological abuse. Such abuse, being (in)visible, was difficult to prove. Thus, because they did not present with visible signs of physical abuse, the women lacked institutional legibility. According to Sweet, legibility is “the ability to be recognized as legitimate and worthy of resources within the institutions” [48]. Lack of institutional legibility here meant missing out on accessing resources. In the current study, the credible victims were women who could provide concrete evidence (discernible marks of physical abuse), who by definition were victims of physical violence. Therefore, the women had to work extremely hard to make themselves legitimate to the available services and support institutions in order to access the available resources. Resources in this study refers to help and support (information and assistance to navigate through, etc.) and being listened to, rather than being questioned and asked to provide evidence that they had indeed experienced psychological abuse. This form of abuse, which is often labelled as “worse” than physical abuse, was generally used to intimidate and to exert control as the “able-bodied” men took advantage of the women’s disability [49].

Psychological abuse being (in)visible and hard to prove, coupled with (dis)ability status, especially intellectual disabilities, meant that the women were faced with suspicion and mistrust when they claimed such abuse. Legibility demands also drove the women away from seeking help. In previous studies, the factors that increased the vulnerability to abuse of women with disabilities included not being trusted, and not being listened to, which applied especially to those with an intellectual disability [6, 11]. Furthermore, women with learning disabilities reported abuse to different services and support institutions, but never received the appropriate help, and such women expressed the desire to be listened to and believed because mistrust had a psychological impact of its own, resulting in experiences of self-blame and powerlessness [20].

The legal system, along with other services and support institutions, seems to have created an array of demands for how victims of abuse should conduct themselves and report the abuse, and these demands shape their institutional experience [48]. In Sweden, the grey literature reveals the existence of current debates about labeling psychological violence as a crime. This indicates that its visibility and provability might improve, thus leading to ‘easy’ institutional legibility. But then the question arises: should psychological abuse be labeled as a crime for it to be legible?

### Continuation of violence from a temporal perspective

In this study, participants described experiences of abuse that had continued from childhood into adolescence and adulthood, and after leaving their abusive relationships. This pattern depicts a life-course trend of violence. Women with disabilities might require help and support from others throughout their life course, depending on the nature of the disability, and this is also a risk factor for multiple forms of abuse from multiple abusers [19] extended over long durations. Some of the reasons for such prolonged abuse described by the participants in this study included social and physical isolation, both by the abusers and initiated by the woman herself (due to feelings of shame), and dependence, which secluded the women from the outside world, and thus prolonged the abuse. Dependence thus led to greater exposure to violence, and women remaining for longer periods in abusive relationships. Leaving abusive partners seemed to be just a “reliever” but did not put an end to the abuse entirely as it often continued beyond separation. The women suffered from what has been described as post-relationship re-victimization, where one experiences subsequent violence even after the relationship has ended [20]. This included psychological abuse persisting, the ex-partners continuing to exert control, financial frustrations, denial of the right to their children, frustration about communication, and making co-parenting unbearable.

### Many ways to survive violence—victims doing something

All the women in this study had left their abusive partners and were leading a different life compared to when they had been in abusive relationships. Importantly, even before leaving, they were all doing something about their situation. These women dealt with pre- and post-abuse consequences in different ways. Before exiting the abusive relationships, the women “accepted” the violence and attempted to make sense of it by offering explanations as to why their partners were abusive in the hope that things might become better. This coping attitude of women tolerating violence is not unique to women with disabilities but is rather widespread among women without reported disabilities in different geographical contexts [50, 51]. “Accepting and tolerating” violence has also been referred to as normalization. According to Lundgren, the normalization process refers to violence that is increasingly perceived by the victim as a normal occurrence in everyday life due to the acceptable limits becoming stretched [52]. In the current study, such normalization and staying longer in the abusive relationships were due to: hopes that the abusers would change; blaming oneself; dependence on the abusers for assistance, the abuser was doing them a “favor” by “loving” and living with a disabled woman; and wanting to uphold the family and societal stereotypes. These were typical descriptions of how relationships should be, especially among women who had experienced psychological abuse. For instance, a study on managing abusive experiences among older women revealed that some of them utilized various strategies, such as self-blame to navigate such abusive experiences [53].

The findings of this study reveal that women do many things to survive; some of these things may be quite useful and healthy, while others might be harmful. It is important to underscore that some survival tactics might have consequences for how the women feel about themselves and might potentially influence the probability that they will seek help. It is imperative to support women in leaving abusive relationships, but at the same time to ensure that they do not feel ashamed or embarrassed for being a victim/survivor of IPV. It is noteworthy to mention that women usually do not stay in abusive relationships willingly. Their choices are often limited by many underlying forces, such as societal stereotypes and prejudices, financial dependence, cultural norms, and specifically dependence on the abuser for help and support in the case of women with disabilities [6].

### Implications

The results of the current study underscore the importance of ensuring that the services and support institutions are more responsible and proactive. This is crucial to enhance screening efforts for IPV among women with disabilities. These findings may also prompt discussions on policies and guidelines regarding how institutions should respond to vulnerability of women with disabilities. To better identify and support women with disabilities who experience IPV, education and sensitization interventions should be developed targeting not only women themselves, but also services and support providers across various institutions. This support should continue even after the women have left abusive relationships (e.g., revisions to custody laws). Additionally, service providers’ capacity should be enhanced to better meet the needs of this population.

## Conclusion

The findings of this study show that women with disabilities experience multiple forms of abuse, including sexual, physical, financial, and psychological abuse. These abuses occur over time and may involve different perpetrators, not only intimate partners. Moreover, the results reveal that women with disabilities often face difficulties in proving psychological abuse, which they perceive as more detrimental than physical violence which often receive greater attention. The results of the study also highlight that abuse does not necessarily end with separation or divorce, as factors such as financial dependence and co-parenting enable the continuation of psychological abuse.

In order to cope with abusive relationships, women employed a range of survival strategies. These strategies include reflecting on their own experiences of violence, engaging in stress-relieving activities, and actively seeking information to better understand their situations.

## Electronic supplementary material

Below is the link to the electronic supplementary material.

Additional file: Interview guide

## Data Availability

The datasets used and/or analyzed during the current study are available from the corresponding author on reasonable request.

## Abbreviations

IPV: Intimate partner violence
WHO: World health organization
RTA: Reflexive thematic analysis
PATH: Program for appropriate technology in health
