# Supplementary material for: Women with disabilities’ experiences of intimate partner violence: a qualitative study from Sweden
Source: BMC Womens Health. 2023 Jul 20;23:381. doi: 10.1186/s12905-023-02524-8 (PMC10360297; doi:10.1186/s12905-023-02524-8)
Supplement: Supplementary file 1 — Additional file: Interview guide [file 12905_2023_2524_MOESM1_ESM.pdf]

Interview guide

The following questions serve as a guide for what to ask during the interviews. They are not intended to be followed in a specific order, and they provide flexibility for additional follow-up questions that may not be listed below.

Introduction

Please tell us a little about yourself and what made you participate in this study?

Accessibility/contact with IPV support and services

- o Give examples of situation(s) when you have sought support and/or help due to intimate partner violence.
- Did you receive support? What kind of support? Describe the content of the support and assistance you were offered.
- Was it easy or difficult to get in touch with the help and support you received? Was it as you thought – or easier or harder?
- Do you know if there are additional activities that offer support and assistance for people with disabilities who have experienced intimate partner violence? Which? What kind of support do they offer? Do you have experience with these?
- Was the information you received about various support and assistance measures relevant to your own needs at the time?
- What kind of long-term support or interventions did you receive? (What form of long-term follow-up or short term are provided?

Quality of IPV support, services and suitability

- How did you experience - or what did you feel - or what feelings did you experience - receiving support and help as a result of intimate partner violence? Relief? Shame? Anger? Uncertainty?
- What is your view of the treatment and quality of the services you have been offered by the following sectors: a) the police, b) social workers, c) medical staff and d) shelters?
- How have you been treated by those who work with support and different activities? (some they already answer in the previous question)

- Do you feel that the efforts you received were helpful to you? Feel free to elaborate.
- Do you feel that the efforts you have received can be helpful to other people who have been through similar situations? Feel free to elaborate.

Competence development and recommendations

- From your point of view, what are the most important areas to focus on when offering support and assistance for people with disabilities who have experienced intimate partner violence?
- Tell us, how do you think these sectors(the ones mentioned above) should and can develop their skills, both in terms of treatment and content in the support efforts?

Covid 19 and intimate partner violence

- What has affected the corona crisis's risk of care for intimate partner violence
  - Did you apply for support/advice due to Covid crisis?
  - How has Covid pandemic affected how you reach support/advice

Conclusion

- Finally, is there anything you expected but we didn 't address? Anything you want to send us in the project?

Thanks!
